# Red cell distribution width and mean platelet volume detection in patients with endometrial cancer and endometrial hyperplasia

**DOI:** 10.1002/hsr2.70109

**Published:** 2024-10-08

**Authors:** Zahra Rafiei Sorouri, Roya Kabodmehri, Forozan Milani, Parmoon Parvari

**Affiliations:** ^1^ Department of Obstetrics & Gynecology, Reproductive Health Research Center, Al‐Zahra Hospital, School of Medicine Guilan University of Medical Sciences Rasht Iran

**Keywords:** endometrial cancer, endometrial hyperplasia, platelet volume, red cell

## Abstract

**Background:**

Endometrial cancer is the most common malignancy in women in developed countries, and its incidence is increasing annually. Due to the availability and cost‐effectiveness of serum markers of red cell distribution width (RDW), and mean platelet volume (MPV), we decided to investigate these two important markers in patients with endometrial cancer and assess their role in diagnosing the tumor and differentiate it from endometrial hyperplasia and other causes of bleeding.

**Methods:**

This is a case‐control study that examined the data of patients who were referred to Al‐Zahra Hospital during 2022–2023 with complaints of abnormal bleeding and underwent diagnostic curettage. Based on the pathology findings, the patients were divided into 3 groups, including endometrial cancer, endometrial hyperplasia, and control. The clinical characteristics and results of MPV and RDW were compared in these three groups. The IBM SPSS Statistics for Windows, Version 21.0. was used for data analysis.

**Results:**

In this study, 87 women were examined in three groups endometrial cancer, endometrial hyperplasia, and control with a mean age of 52.70 ± 11.63 years. The results showed that the endometrial cancer group, had higher gravida, underlying disease, history of radiation therapy, anticoagulant therapy, blood transfusion, surgery, and family history of cancer (*p* < 0.05). Meanwhile, the endometrial cancer group had lower menstrual age and history of using contraceptives than other groups (*p* < 0.05). In addition, in this study, the results indicated that the levels of MPV and RDW in the endometrial cancer group were significantly higher than in the endometrial hyperplasia and control groups (*p* < 0.05).

**Conclusion:**

Since MPV and RDW are cheap and accessible and can be easily obtained from complete blood count panels, they can be used as suitable diagnostic markers for endometrial cancer. However, conducting comprehensive multicenter prospective studies with a larger sample size can be helpful.

## 1 INTRODUCTION

Endometrial cancer is the most common malignancy in women in developed countries, and its incidence is increasing annually.^1^ In 2018, 121,578 new cases of this malignancy were reported in Europe, of which 29,638 died, and its incidence was associated with increasing age and obesity.^2^ Although its definite cause is unknown, several risk factors have been identified, such as chronic stimulation of the endometrium by estrogen, estrogen replacement, early menarche or late menopause, infertility, lack of pregnancy and ovulation, estrogen‐producing tumors, and demographic characteristics such as old age, white race, good social and economic status, family history of endometrial cancer, and the presence of co‐morbidities such as diabetes, gall bladder diseases, obesity, blood pressure, and history of pelvic radiotherapy.^3^, ^4^, ^5^ The importance of fertility‐sparing treatments, especially for young patients desiring future pregnancies, has gained significant attention in recent years. Oocyte vitrification has emerged as a crucial option for preserving fertility in patients undergoing treatment for endometrial cancer. Incorporating this strategy into patient management can help address the growing need for personalized treatment plans.

Besides, early diagnosis of this disease is very effective in determining the preferred treatment method and timely action. Previous investigations have been conducted on serum biomarkers that can lead to the clinician's suspicion towards this diagnosis including circulating miRNAs, which are emerging as powerful tools for early diagnosis.^6^, ^7^, ^8^ Among these biomarkers, Red cell distribution width (RDW) and mean platelet volume (MPV) have been highlighted for their potential roles in predicting and staging endometrial cancer.^7^, ^8^ Additionally, recent studies have shown that a combination of RDW and CA125 can serve as a strong prognostic factor for endometrial cancer. One of these markers is RDW, whose mechanism in tumors is the presence of underlying inflammation, nutritional disorders, and oxidative stress.^8^ The next marker is the MPV, which is automatically measured in blood tests. A previous study showed that MPV and RDW are useful biomarkers for predicting endometrial cancer.^9^ Also, in another study, they found a relationship between the number of RDW and MPV with the stage of cancer.^6^ It is worth noting that in a recent study, it was reported that the simultaneous combination of two markers, RDW and CA125, is a strong factor in determining the prognosis of endometrial cancer.^7^

As endometrial tumors have genetic polymorphism and a specific molecular nature,^10^, ^11^ their accurate diagnosis and prognosis are still under discussion, and introducing markers that can rely on their diagnostic role will have a significant impact on determining the course of treatment. Considering the availability and affordability of the mentioned markers, we decided to investigate these two important markers (RDW, MPV) in patients with endometrial cancer and their role in diagnosing this tumor and distinguishing it from endometrial hyperplasia.

## 2 MATERIALS AND METHODS

### 2.1 Study design

This is a case‐control study that was done by the convenient sampling method from August 2022 to January 2023.

### 2.2 Study population

All the patients undergoing diagnostic curettage due to abnormal uterine bleeding were referred to Al‐Zahra Hospital, Iran.

The ethical approval was obtained from the ethics committee of the Vice‐Chancellor of Research at Guilan University of Medical Sciences (code: IR. GUMS. REC.1401.254, date: 2022‐07‐20).

### 2.3 Study groups

Patients were divided into three groups, including endometrial cancer, endometrial hyperplasia, and control based on the pathology findings. Controls were those who were healthy in pathology results.

Inclusion criteria were not receiving previous treatment, blood sampling before biopsy, absence of diagnostic lesion, or significant pathology. The exclusion criteria were the presence of any underlying diseases such as blood diseases, malignancies, chronic inflammations, diabetes, kidney, and cardiovascular disorders, anemia, blood transfusion in the previous 3 months, and vascular thrombosis during the previous 6 months, hyperprolactinemia, abnormal thyroid tests, recent use of anticoagulants, and hormone therapy during the past year.

Data were gathered by a form, including age, gravida, parity, age of menarche, age of menopause, underlying diseases (e.g., high blood pressure, liver‐biliary diseases, thyroid disorders, heart disease, lung disease, allergy, and neurological disease), history of chemotherapy or radiation therapy, taking anticoagulants, blood transfusion, using contraceptives or assisted reproductive technologies, surgery, and family history of cancer. RDW, MPV, and pathology results were also assessed.

### 2.4 Sample size and sampling methods

The sample size was indicated using Zhang et al. The formula estimated the sample size of 29 for each group.^6^

$$n=\frac{{\left(z_{1-\frac{\alpha }{2}}+z_{1-\beta }\right)}^{2}(\sigma_{2}^{2}+\sigma_{1}^{2})}{{\left(\mu_{2}-\mu_{1}\right)}^{2}}$$

α=0.05β=0.15

$${µ}_{1\;\pm }\;\sigma_{1}=\;11.51\;\pm 2.22$$

$$\mu_{2}+\;\sigma_{2}=9.58\;\pm 1.84$$

### 2.5 Statistical analysis

The IBM SPSS Statistics for Windows, Version 21.0. was used for data analysis. Mean and standard deviation were used to describe quantitative data, and number and percent were used to describe qualitative data. The normality of the data was checked using the Shapiro‐Wilk test. To check the relationship between the data, and if the data were normal, the ANOVA test was used. The Kruskal‐Wallis test was used if the data were non‐normal. Post hoc tests (Tuckey and Mann–Whitney U) were used to check the difference between the two groups. The significance level was considered to be less than 0.05 (*p*‐value < 0.05).

## 3 RESULTS

In this study, 87 women were examined in three groups of 29, including endometrial cancer, endometrial hyperplasia, and control, with a mean age of 52.70 ± 11.63 years. Among the investigated quantitative variables, only the menopausal age and MPV variables had a normal distribution, and to compare them in the three groups, ANOVA parametric test was used. Other quantitative variables had a non‐normal distribution and the Kruskal‐Wallis test was used to compare three groups.

As Table 1 shows, The Kruskal‐Wallis test showed a significant difference in terms of age in the three groups (*p* < 0.001). The results of the Mann–Whitney post hoc test, to check the difference between the groups, also indicated that the age in the endometrial cancer group was significantly higher than both the endometrial hyperplasia (*p* < 0.001) and the control (*p* < 0.001) groups, while the age in the endometrial hyperplasia group was not significantly different from the control group (*p* = 0.895).

**Table 1:** Comparing groups in terms of demographic and clinical results.

Variables	Groups			*P*‐value
	Endometrial cancer	Endometrial hyperplasia	Control	
Age Median (interquartile range)	63 (57–68)	46 (39.5–52)	45 (40.5–55.0)	<0.001*
Parity Median (interquartile range)	2 (1–4)	3 (1–3)	2 (1–3)	0.211*
Gravida Median (interquartile range)	4 (2–6)	3 (1–3)	2 (2–3)	0.001*
Age at menarche Median (interquartile range)	11 (11–12)	13 (11–13)	12 (11–13)	0.001*
Menopause age Mean (standard deviation)	49.86 (2.90)	49.25 (1.58)	49.17 (1.19)	0.648**
Underlying diseases				<0.001***
Yes num (%)	28 (96.6%)	12(41.4%)	12(41.4%)	
No num (%)	1(3.4%)	17(58.6%)	17(58.6%)	
Chemotherapy				0.063***
Yes num (%)	4 (13.8%)	1(3.4%)	0 (0%)	
No num (%)	25 (86.2%)	28 (96.6%)	29 (100%)	
Radiotherapy				0.045***
Yes num (%)	3(10.3%)	0 (0%)	0 (0%)	
No num (%)	26 (89.7%)	29 (100%)	29 (100%)	
Anticoagulant therapy				0.015***
Yes num (%)	7 (24.1%)	3(10.3%)	0 (0%)	
No num (%)	22 (75.9%)	26 (89.7%)	29 (100%)	
Blood transfusion				<0.001***
Yes num (%)	16 (55.2%)	4(13.8%)	2(6.9%)	
No num (%)	13(44.8%)	25 (86.2%)	27 (93.1%)	
Contraceptive use				0.023***
Yes num (%)	6 (20.7%)	16 (55.2%)	10 (34.5%)	
No num (%)	23 (79.3%)	13 (44.8%)	19 (65.5%)	
Assisted reproductive technology				0.313***
Yes num (%)	4 (13.8%)	6 (20.7%)	2 (6.9%)	
No num (%)	25 (86.2%)	23 (79.3%)	27 (93.1%)	
Surgery				<0.001***
Yes num (%)	26 (89.7%)	14 (48.3%)	12(41.4%)	
No num (%)	3(10.3%)	15 (51.7%)	17(58.6%)	
Family history of cancer				0.023***
Yes num (%)	9 (31.0)	1 (3.4%)	6 (20.7%)	
No num (%)	20 (69%)	28 (96.6%)	23 (79.3%)	

* Kruskal‐wallis test,

** ANOVA test,

*** Chi‐square test, *p*‐value < 0.05

Also, there was no significant difference in terms of parity in the three groups (*p* = 0.211). The results of the post hoc test indicated that parity in the endometrial cancer group was not significantly different from the endometrial hyperplasia group (*p* = 0.274) and control (*p* = 0.163) and no significant difference was observed between the endometrial hyperplasia group with the control group (*p* = 0.636).

However, the results showed a significant difference between the three groups in terms of gravida (*p* = 0.001). It was found that the gravida in the endometrial cancer group was significantly higher than the endometrial hyperplasia group (*p* = 0.023) and the control (*p* = 0.009), while the gravida in the endometrial hyperplasia group was not significantly different from the control group (*p* = 0.542).

In addition, there was a significant difference between the three groups in terms of age at menarche (*p* = 0.001). The results of the post hoc test indicated that it was higher in the endometrial cancer group than both the hyperplasia (*p* = 0.020) and the control groups (*p* = 0.011), while the age of menarche in the endometrial hyperplasia group was not significantly different from the control group (*p* = 0.975).

In this study, there was no significant difference in the three groups in terms of menopause age (*p* = 0.648). The results of the post hoc test also indicated that the age of menopause in the endometrial cancer group was significantly different from the endometrial hyperplasia group (*p* = 0.514) and the control group (*p* = 0.395) and no difference was observed in the endometrial hyperplasia group with the control group (*p* = 0.605).

There was no significant difference in the three groups in terms of the history of chemotherapy (*p* = 0.063), while there was a significant difference in the three groups regarding the history of radiation therapy (*p* = 0.045), such that 10.3% of patients in the endometrial cancer group had a history of radiation therapy. Besides, there was a significant difference in the three groups in terms of the history of anticoagulant therapy, such that 24% in the endometrial cancer group and 10.3% in the hyperplasia group, had a history of anticoagulant therapy (*p* = 0.015). Although there was a significant difference in the three groups in terms of the history of blood transfusion (*p* < 0.001), contraceptive use (*p* = 0.023), surgery (*p* > 0.001), and family history of cancer (*p* = 0.023), no significant difference was noted regarding the use of assisted reproductive technology (*p* = 0.313) (Table 1).

Our results showed a significant difference in terms of mean MPV in the three groups (*p* < 0.001). The results of Tukey's post hoc test indicated that the mean MPV in the endometrial cancer group was significantly higher than both endometrial hyperplasias (*p* < 0.001) and control (*p* < 0.001) groups and in the endometrial hyperplasia was significantly higher than the control group (*p* < 0.001). In addition, there was a significant difference in terms of the RDW level in the three groups, so the RDW level in the endometrial cancer group was significantly higher than in the endometrial hyperplasia and control groups (*p* > 0.001). The results of the Mann‐Whitney post hoc test also indicated that the RDW in the endometrial cancer group was significantly higher than both the endometrial hyperplasia group (*p* < 0.001) and the control group (*p* < 0.001) and in the endometrial hyperplasia was significantly higher than the control group (*p* < 0.001) (Table 2).

**Table 2:** Comparing groups regarding MPV and RDW.

Variables	Groups			*P*‐value
	Endometrial cancer	Endometrial hyperplasia	Control	
MPV mean (SD)	11.28 (1.37)	9.64 (1.07)	7.63 (0.59)	<0.001*
RDW Median (interquartile range)	16.80 (15.55–17.55)	14.20 (13.10–14.85)	12.20 (11.90–12.95)	<0.001**

* ANOVA,

** Kruskal‐wallis test, *p*‐value < 0.05

## 4 DISCUSSION

Endometrial cancer is the most common malignancy among women's neoplasms, the mechanism of which has not been fully determined yet. In this study, three groups of women with endometrial cancer, endometrial hyperplasia, and a control group were examined. The results showed that the endometrial cancer group had significantly higher gravida, underlying disease, history of radiation therapy, anticoagulant therapy, blood transfusion, surgery, and family history of cancer. Meanwhile, the endometrial cancer group had lower menstrual age and used contraceptives than other groups. In addition, in this study, the results indicated that the levels of MPV and RDW in the endometrial cancer group were significantly higher than in the endometrial hyperplasia and control groups.

As it was found in this study, women with endometrial cancer were significantly older than other groups, and similar to the present study, the study by Yayla Abide et al. also indicated that the group with endometrial cancer was older. It is worth noting that the older age in people with cancer is because estrogen production stops after menopause and most of the estrogens in the blood circulation are produced as a result of the environmental aromatization of androgens to estrogen in fat tissue. Therefore, it has been found that pro‐inflammatory cytokines can stimulate aromatase activity in adipose tissue and thus increase estrogen production and bioavailability. This situation explains why endometrial carcinoma is more common in older and obese women than in others.^9^

In previous studies, the results showed that diabetes and high blood pressure are diseases that increase the risk of endometrial cancer. As Zhao et al. reported, obesity‐related metabolic syndrome was considered responsible for the increased risk of endometrial cancer due to insulin resistance, limited physical activity, low high‐density lipoprotein levels, high triglyceride levels, diabetes mellitus, and hypertension.^12^ In another study, it was found that the frequency of diabetes and high blood pressure in the endometrial cancer group was significantly higher than in the endometrial hyperplasia group and the control group.^13^ Of course, in our study, there was a significant difference in the three groups in terms of underlying disease, such that most people in the endometrial cancer group had the underlying disease (96.6%), but the type of disease was not investigated.

Platelet count and MPV are the most important parameters indicating platelet activity. An increase in the level of MPV indicates the presence of large and active platelets in the peripheral circulation. An increase in MPV levels is considered a consequence of systemic inflammatory response, which plays an important role in the development and progression of cancer. It has also been found that MPV is a useful indicator in some inflammatory diseases, which is related to disease activity and inflammation severity.^14^ Studies have shown the role of decreased MPV levels in non‐small cell lung cancer, multiple myeloma, and primary dysmenorrhea.^15^, ^16^, ^17^ Regarding the possible reduction of MPV, it has been determined that released inflammatory mediators increase the activation of platelets, which leads to subsequent changes in MPV. Platelet consumption at the site of inflammation may be responsible for the reduction in MPV. In addition, the inflammatory environment can damage megakaryopoiesis and in turn, cause the release of small platelets from the bone marrow.^18^ However, so far studies have shown an increase in peripheral blood MPV levels in different types of cancer, such as liver cancer and ovarian cancer, and breast cancer.^18^, ^19^

In this study, it was also found that the level of MPV in the cancer group was significantly higher than in other groups. Similar to the present study, in the study of Abide et al, the results indicated that the preoperative MPV measurement in the endometrial cancer group and the endometrial hyperplasia group was significantly higher than the control group.^9^ In addition, in the study of Karateke et al., which was also conducted in patients with endometrial cancer and endometrial hyperplasia, the highest MPV value was determined in the endometrial carcinoma group, and the lowest MPV value was determined in the control group.^20^ The difference in the mentioned results of the increase and decrease of this parameter in the past studies, maybe due to the difference in the type of diseases, and the methodological characteristics and participants in the studies.

Another inflammatory marker, RDW is a marker of heterogeneity in the size of circulating red blood cells and is used to determine the degree of anisocytosis in peripheral blood.^21^ RDW is an indicator of erythropoiesis disorder and abnormal survival of red blood cells. It has been shown that inflammatory cytokines play a role in inhibiting the stimulatory effect of erythropoietin on bone marrow erythrocyte stem cells, antiapoptosis, and cell maturation, thus causing more immature erythrocytes to be released into the peripheral circulation and as a result, It increases the heterogeneity of peripheral red blood cells and RDW.^22^ An increase in RDW may also be associated with an increase in ineffective hematopoiesis due to chronic inflammation. The study of Bennet et al. showed that the inflammatory response and oxidative stress can affect erythropoiesis and change the plasticity of cell membranes.^23^

In recent years, there have been studies investigating the relationship between RDW and cardiovascular diseases and cancer. RDW is an independent prognostic factor for many cancers such as lung cancer, prostate cancer, and chronic lymphocytic leukemia^24^, ^25^, ^26^ In our study, the results indicated that the level of this factor was significantly higher than in other groups. However, in the study of Yayla Abide et al., the level of RDW in patients with endometrial carcinoma was significantly lower than that of endometrial hyperplasia and healthy individuals.^9^ These conflicting results could be due to different sample sizes and indicate the need for further investigations. Besides, it should be noted that in a meta‐analysis conducted on a limited number of tumors to determine the prognostic value of RDW in cancer patients, Hu et al. concluded that increased RDW is an unfavorable predictor in cancer patients.^27^

### 4.1 Strengths and limitations

While this research compared three groups of patients in terms of important parameters, it had some limitations. The small sample size and the retrospective nature of this study in cancer patients are the most important limitations of this study. Likewise, not examining other inflammatory factors and evaluating their relationship with the main facts in this study could have been helpful. Furthermore, recent advances in molecular and genomic profiling have identified novel prognostic factors in endometrial cancer, such as L1CAM.^28^, ^29^ Therefore, further investigations highlighting the importance of incorporating molecular markers into clinical practice to refine prognostic assessments and personalized treatments can be recommended.

## 5 CONCLUSIONS

So far, other inflammatory markers have been investigated to identify cases of endometrial cancer, such as CRP, IL6, and IL1Ra, and a significant relationship has been identified between these markers and endometrial cancer.^30^ However, MPV and RDW, which are inexpensive and accessible, and easily obtained from complete blood count panels, can be used as suitable diagnostic markers for endometrial cancer. However, conducting more comprehensive studies with a larger sample size in a multicenter and prospective manner can be helpful.

## AUTHOR CONTRIBUTIONS

**Zahra Rafiei Sorouri**: Conceptualization; validation; writing—original draft; visualization; investigation. **Roya Kabodmehri**: Project administration conceptualization; writing—review and editing; resources. **Forozan Milani**: Methodology; validation; data curation; formal analysis. **Parmoon Parvari**: Writing—original draft; data curation; formal analysis; project administration; software; investigation; methodology; writing—review & editing.

## CONFLICT OF INTEREST STATEMENT

The authors declare no conflicts of interest.

## FUNDING

This study was financially supported by the Vice‐Chancellorship of Research and Technology, Guilan University of Medical Science. The authors declare that there is no conflict of interest.

## ETHICS STATEMENT

The ethics committee of Guilan University of Medical Sciences approved the survey (IR. GUMS. REC.1401.254). All cases filled out the written informed consent before the study begins.

All information in this article has a code of ethics and is approved by the Department of Obstetrics & Gynecology, Al‐Zahra Hospital, School of Medicine, Guilan University of Medical Sciences, Rasht, Iran.

Human Participants: Ethical approval was obtained from the ethics committee of Guilan University of Medical Sciences (Approval ID: IR. GUMS. REC. 1401.254). Informed consent was taken from all participants. All stages of this research have been performed according to the Helsinki declaration. All procedures of the study were explained clearly to the participants who had the eligible inclusion criteria. Moreover, all participants voluntarily filled out the written informed consent form before they join the study and they were free to decide whether or not to attend or withdraw at any time and for any reason without changing the medical care.

## TRANSPARENCY STATEMENT

The lead author Roya Kabodmehri affirms that this manuscript is an honest, accurate, and transparent account of the study being reported; that no important aspects of the study have been omitted; and that any discrepancies from the study as planned (and, if relevant, registered) have been explained.

## INFORMED CONSENT

The written informed consent letter was taken from all participants.

## Data Availability

The data that support the findings of this study are available on request from the corresponding author. The data are not publicly available due to privacy or ethical restrictions. All authors have read and approved the final version of the manuscript. Corresponding author had full access to all of the data in this study and takes complete responsibility for the integrity of the data and the accuracy of the data analysis. supporting data are available in the Reproductive Health Research Center, Department of Obstetrics & Gynecology, Al‐Zahra Hospital, School of Medicine, Guilan University of Medical Sciences, Rasht, Iran.

## References

[1] Mutlu L , Manavella DD , Gullo G , McNamara B , Santin AD , Patrizio P . Endometrial cancer in reproductive age: fertility‐sparing approach and reproductive outcomes. Cancers. 2022;14:5187. 36358604 10.3390/cancers14215187 PMC9656291

[2] Clarke MA , Long BJ , Del Mar Morillo A , Arbyn M , Bakkum‐Gamez JN , Wentzensen N . Association of endometrial cancer risk with postmenopausal bleeding in women: a systematic review and meta‐analysis. JAMA Intern Med. 2018;178:1210–1222. 30083701 10.1001/jamainternmed.2018.2820 PMC6142981

[3] Lee YC , Lheureux S , Oza AM . Treatment strategies for endometrial cancer: current practice and perspective. Curr Opin Obstet Gynecol. 2017;29:47–58. 27941361 10.1097/GCO.0000000000000338

[4] Giampaolino P , Cafasso V , Boccia D , et al. Fertility‐sparing approach in patients with endometrioid endometrial cancer grade 2 stage IA (FIGO): a qualitative systematic review. BioMed Res Int. 2022;2022:1–15. 10.1155/2022/4070368 PMC9532104 36203482

[5] Gullo G , Perino A , Cucinella G . Open vs. closed vitrification system: which one is safer? Eur Rev Med Pharmacol Sci. 2022;26:1065–1067. 35253158 10.26355/eurrev_202202_28092

[6] Piergentili R , Gullo G , Basile G , et al. Circulating miRNAs as a tool for early diagnosis of endometrial cancer—Implications for the Fertility‐Sparing process: clinical, biological, and legal aspects. Int J Mol Sci. 2023;24:11356. 37511115 10.3390/ijms241411356 PMC10379073

[7] Zhong W , Zhou C , Chen L , et al. The coefficient of variation of red blood cell distribution width combined with cancer antigen 125 predicts postoperative overall survival in endometrial cancer. Int J Gen Med. 2021;14:5903–5910. 34584444 10.2147/IJGM.S323136 PMC8464372

[8] Zhang H , Liang K , Ke L , Tang S . Clinical application of red cell distribution width, mean platelet volume, and cancer antigen 125 detection in endometrial cancer. J Clin Lab Anal. 2020;34:e23309. 32196750 10.1002/jcla.23309 PMC7439417

[9] Yayla Abide C , Bostanci Ergen E , Cogendez E , et al. Evaluation of complete blood count parameters to predict endometrial cancer. J Clin Lab Anal. 2018;32:e22438. 29604099 10.1002/jcla.22438 PMC6817014

[10] Raffone A , Travaglino A , Gabrielli O , et al. Clinical features of ProMisE groups identify different phenotypes of patients with endometrial cancer. Arch Gynecol Obstet. 2021;303:1393–1400. 33754186 10.1007/s00404-021-06028-4 PMC8087601

[11] Gullo G , Cucinella G , Chiantera V , et al. Fertility‐sparing strategies for early‐stage endometrial cancer: stepping towards precision medicine based on the molecular fingerprint. Int J Mol Sci. 2023;24(1):811. 36614253 10.3390/ijms24010811 PMC9821405

[12] Zhao J , Hu Y , Zhao Y , Chen D , Fang T , Ding M . Risk factors of endometrial cancer in patients with endometrial hyperplasia: implication for clinical treatments. BMC Womens Health. 2021;21:312. 34433451 10.1186/s12905-021-01452-9 PMC8390278

[13] Yang X , Wang J . The role of metabolic syndrome in endometrial cancer: a review. Front Oncol. 2019;9:744. 31440472 10.3389/fonc.2019.00744 PMC6694738

[14] Korniluk A , Koper‐Lenkiewicz OM , Kamińska J , Kemona H , Dymicka‐Piekarska V . Mean platelet volume (MPV): new perspectives for an old marker in the course and prognosis of inflammatory conditions. Mediators Inflamm. 2019;2019:1–14. 10.1155/2019/9213074 PMC6501263 31148950

[15] Gao L , Zhang H , Zhang B , Zhang L , Wang C . Prognostic value of combination of preoperative platelet count and mean platelet volume in patients with resectable non‐small cell lung cancer. Oncotarget. 2017;8:15632–15641. 28152504 10.18632/oncotarget.14921 PMC5362511

[16] Zhuang Q , Xiang L , Xu H , et al. The Independent association of mean platelet volume with overall survival in multiple myeloma. Oncotarget. 2016;7:62640–62646. 27566590 10.18632/oncotarget.11551 PMC5308753

[17] Kabil Kucur S , Seven A , Yuksel KB , Sencan H , Gozukara I , Keskin N . Mean platelet volume, a novel biomarker in adolescents with severe primary dysmenorrhea. J Pediatr Adolesc Gynecol. 2016;29:390–392. 26876966 10.1016/j.jpag.2016.01.128

[18] Detopoulou P , Panoutsopoulos GI , Mantoglou M , et al. Relation of mean platelet volume (MPV) with cancer: a systematic review with a focus on disease outcome on twelve types of cancer. Curr Oncol. 2023;30:3391–3420. 36975471 10.3390/curroncol30030258 PMC10047416

[19] Lu L , Su Z , Zheng P , et al. Association between platelet count and hepatocellular carcinoma overall survival: a large retrospective cohort study. BMJ Open. 2020;10(11):e038172. 10.1136/bmjopen-2020-038172 PMC7651714 33158820

[20] Karateke A , Kaplanoglu M , Baloglu A . Relations of platelet indices with endometrial hyperplasia and endometrial cancer. Asian Pacific J Cancer Prevent. 2015;16:4905–4908. 10.7314/apjcp.2015.16.12.4905 26163613

[21] Paschos KatsarosM , Giouleme P . O. Red cell distribution width as a marker of activity in inflammatory bowel disease: a narrative review. Ann Gastroenterol. 2020;33:348–354. 32624654 10.20524/aog.2020.0486 PMC7315702

[22] Owoicho O , Tapela K , Olwal CO , Djomkam Zune AL , Nganyewo NN , Quaye O . Red blood cell distribution width as a prognostic biomarker for viral infections: prospects and challenges. Biomark Med. 2022;16:41–50. 34784758 10.2217/bmm-2021-0364 PMC8597662

[23] Bennett LF , Liao C , Quickel MD , et al. Inflammation induces stress erythropoiesis through heme‐dependent activation of SPI‐C. Sci Signal. 2019;12:eaap7336. 31506384 10.1126/scisignal.aap7336 PMC6904953

[24] Ai L , Mu S , Hu Y . Prognostic role of RDW in hematological malignancies: a systematic review and meta‐analysis. Cancer Cell Int. 2018;18:61. 29713244 10.1186/s12935-018-0558-3 PMC5914059

[25] Mao XL , Xi YM , Li ZJ , et al. Higher red blood cell distribution width at diagnose is a simple negative prognostic factor in chronic phase‐chronic myeloid leukemia patients treated with tyrosine kinase inhibitors: a retrospective study. Medicine. 2021;100:e24003. 33725811 10.1097/MD.0000000000024003 PMC7969257

[26] Eoh KJ , Lee TK , Nam EJ , Kim SW , Kim YT . Clinical relevance of red blood cell distribution width (RDW) in endometrial cancer: a retrospective Single‐Center experience from korea. Cancers. 2023;15:3984. 37568799 10.3390/cancers15153984 PMC10417026

[27] Hu L , Li M , Ding Y , et al. Prognostic value of RDW in cancers: a systematic review and meta‐analysis. Oncotarget. 2017;8:16027–16035. 27926498 10.18632/oncotarget.13784 PMC5362543

[28] Giannini A , D'Oria O , Corrado G , et al The role of L1CAM as predictor of poor prognosis in stage I endometrial cancer: a systematic review and meta‐analysis. Arch Gynecol Obstet. 2024;309:789–799. 37454351 10.1007/s00404-023-07149-8

[29] Vizza E , Bruno V , Cutillo G , et al. Prognostic role of the removed vaginal cuff and its correlation with L1CAM in low‐risk endometrial adenocarcinoma. Cancers. 2021;14(1):34. 35008194 10.3390/cancers14010034 PMC8750504

[30] Borghi C , Indraccolo U , Scutiero G , et al. Biomolecular basis related to inflammation in the pathogenesis of endometrial cancer. Eur Rev Med Pharmacol Sci. 2018;22:6294–6299. 30338797 10.26355/eurrev_201810_16038

